# MFS‐Unet: A Multi‐Path Vision Mamba Network for Precise Thyroid Nodule Segmentation

**DOI:** 10.1049/syb2.70044

**Published:** 2026-02-05

**Authors:** Shaoqiang Wang, Zhongran Liu, Guiling Shi, Chengye Li, Linhao Zhang, Tiyao Liu, Yawu Zhao, Yuchen Wang, Qiang Li, Xiaochun Cheng

**Affiliations:** ^1^ Qingdao University of Technology Qingdao Shandong China; ^2^ China University of Petroleum (East China) Qingdao China; ^3^ School of Medical Informational Engineering Shandong University of Traditional Chinese Medicine Jinan Shandong China; ^4^ Peking University People's Hospital Qingdao Hospital Qingdao China; ^5^ Swansea University Swansea Wales UK

**Keywords:** biological techniques, biomedical optical imaging, label rectification, multi‐path vision mamba, thyroid nodule segmentation

## Abstract

The automated segmentation of thyroid nodules from ultrasound images holds significant value in clinical diagnosis and treatment. However, achieving precise segmentation remains a substantial challenge due to issues such as blurred nodule boundaries, variable scales, image noise, and inaccurate annotations. To address these difficulties, this paper proposes a novel medical image segmentation network named MFS‐Unet. The network introduces three innovative modules to enhance segmentation performance. First, we designed the multi‐path vision mamba (MPV) module, which leverages the advantages of state space models (SSMs) to efficiently capture global contextual information and multi‐scale features with linear computational complexity, effectively addressing the problem of significant variations in nodule size. Second, a feature gating (FG) module is deployed in the skip connections between the encoder and decoder. Through an attention mechanism, it dynamically screens and enhances features transmitted from the encoder, suppressing background noise and reinforcing key boundary information of the nodules. Finally, we propose a supervised label rectification (SLR) module, aimed at proactively handling the prevalent issue of label noise in training data. By dynamically adjusting loss weights during training, it guides the model to learn more robust feature representations. We conducted extensive experiments on three public thyroid ultrasound datasets: DDTI, TG3K, and TN3K. The results demonstrate that MFS‐Unet achieves superior performance across all evaluation metrics compared with various state‐of‐the‐art segmentation methods, proving its effectiveness and significant potential for precise thyroid nodule segmentation in complex ultrasound environments.

## Graphical abstract

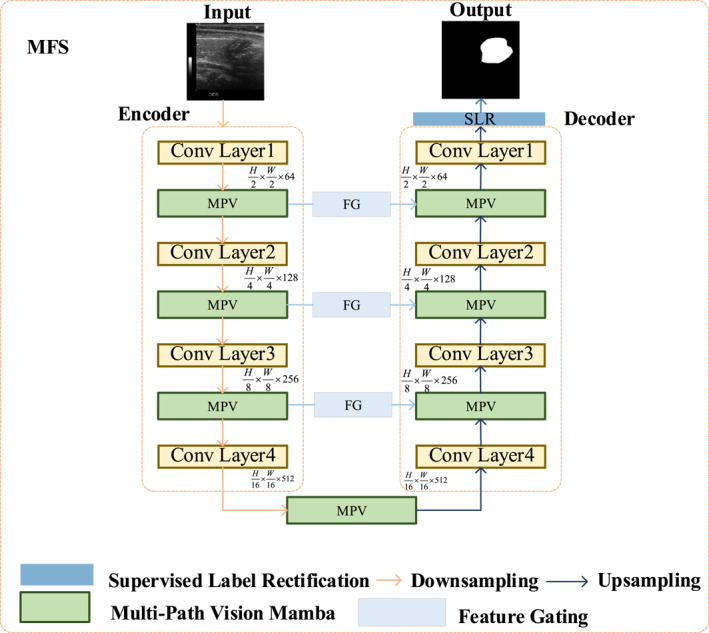

## 1 Introduction

Thyroid nodules are an exceedingly common endocrine system disorder encountered in clinical practice, with their detection rate rising significantly alongside the widespread application of high‐frequency ultrasound technology. Statistically, up to 50% of adults may be found to have thyroid nodules via ultrasound examination, of which approximately 5%–15% are malignant tumours. Consequently, the accurate diagnosis and differentiation of these nodules are crucial for formulating subsequent clinical treatment plans, avoiding unnecessary surgical interventions, and alleviating the physiological and psychological burden on patients.

Among the various imaging technologies, ultrasound imaging has become the preferred modality for evaluating thyroid nodules due to its unique advantages of being non‐invasive, radiation‐free, real‐time, and cost‐effective. However, the clinical interpretation of ultrasound images faces numerous inherent challenges. First, the quality of ultrasound images is highly dependent on the operator's scanning technique and clinical experience, leading to inter‐operator [1] variability. Second, the images themselves are often accompanied by speckle noise, acoustic shadowing, and depth artefacts caused by tissue attenuation, all of which severely interfere with the detailed observation of nodules [2]. More critically, the pathological characteristics of thyroid nodules are extremely complex: their boundaries can be indistinct and difficult to differentiate from surrounding normal tissue; they exhibit diverse morphologies, ranging from regular circular shapes to irregular, spiculated forms; and their sizes span a vast range, from tiny micro‐nodules of a few millimetres to large masses several centimetres in diameter.

These complex imaging characteristics pose a significant challenge to precise medical image segmentation. In the traditional clinical workflow, physicians typically need to delineate the nodule contours for assessment manually. This process is not only time‐consuming and laborious but also subject to significant inter‐observer and intra‐observer variability due to subjective factors. Given this, the development of a high‐precision, high‐robustness automated segmentation method is of irreplaceable clinical value and urgent practical necessity for achieving standardised malignancy assessment (e.g., the thyroid imaging reporting and data system, TI‐RADS), accurate lesion volume measurement, and long‐term dynamic monitoring of disease progression.

In recent years, deep learning methods, spearheaded by U‐Net [3] and its variants, have achieved revolutionary success in the field of medical image segmentation, heralding a new era. These models, based on convolutional neural networks (CNNs), have demonstrated outstanding performance in extracting rich multi‐scale local features and have become the benchmark methods in various segmentation tasks [4]. However, the inherent limitations of CNNs have also become increasingly apparent: their receptive fields are constrained by the size of stacked convolutional kernels, making it difficult for them to effectively capture global contextual information and long‐range pixel dependencies [5]. This is particularly detrimental when dealing with large or irregularly shaped nodules that require extensive contextual information to define their ambiguous boundaries, often leading to discontinuous or incomplete segmentation results.

To overcome this bottleneck, subsequent researchers have drawn inspiration from the vision transformer (ViT) [6] model from the field of natural language processing, utilising its self‐attention mechanism to address long‐range dependency issues [7]. Although ViT and its variants have achieved success in specific tasks, the quadratic computational complexity of their self‐attention modules makes them prohibitively expensive when processing high‐resolution medical images. Furthermore, the ‘data‐hungry’ nature of transformer models presents challenges of difficult training and overfitting in the medical imaging domain, which typically has limited sample sizes [8]. Recently, the emergence of state space models (SSMs) [9], particularly Mamba, has provided a new avenue for efficiently modelling long‐range sequential dependencies, enabling a global receptive field comparable to that of transformers while maintaining linear computational complexity [10].

To address the aforementioned challenges, this paper proposes a novel medical image segmentation network named MFS‐Unet. The network is designed to organically integrate the efficiency of long‐range dependency modelling with the precision of local feature extraction through an innovative architectural design, thereby significantly enhancing the segmentation accuracy and generalisation capability for thyroid nodules in complex ultrasound images [11]. Our main contributions can be summarised in the following three points:

We have designed the multi‐path vision [12] Mamba [13] module (MPV) to replace conventional convolutional blocks in CNNs. This module leverages the powerful capability of SSMs to efficiently model long‐range dependencies with linear complexity. By processing feature maps of different scales in multiple parallel paths, the MPV not only effectively captures global contextual information but also significantly enhances the model's ability to adapt to and process nodules of varying sizes [14].

We have introduced the feature gating module (FG), which serves as a dynamic feature selection unit deployed in the skip connections between the encoder and decoder. Through an attention mechanism, it adaptively evaluates and filters the features passed from the encoder, thereby suppressing irrelevant noise and artefacts from background regions and strengthening the transmission of key nodule features (such as edges and textures), ultimately achieving more transparent and more precise boundary segmentation [15].

We have proposed a supervised label rectification module (SLR) to proactively address the common issue of label noise in medical datasets during the training process. It is important to clarify that in this context, ‘label noise’ does not refer to gross annotation errors. Rather, it pertains to the subtle yet significant challenge of boundary ambiguity arising from two sources: the inherent fuzziness of nodule edges in ultrasound images and the slight inter‐observer variability that exists even among expert physicians [16]. Due to these factors, the ground‐truth masks, while highly accurate, may not represent a single, perfect pixel‐level truth. The SLR module is designed to identify these regions of high uncertainty where the model's learnt predictions and the provided labels are inconsistent. By dynamically adjusting loss weights in these ambiguous areas, it guides the model to learn a more robust and generalised representation of the true physiological boundaries, thereby improving its performance on real‐world imprecisely delineated data [17].

Based on these three innovative modules, we have constructed the complete MFS‐Unet framework. To comprehensively and systematically validate its effectiveness, we have conducted extensive experiments on three challenging public thyroid ultra‐sound datasets (DDTI, TG3K, and TN3K). The experimental results compellingly demonstrate that our model achieves state‐of‐the‐art (SOTA) performance across all key evaluation metrics compared with various advanced segmentation methods, fully showcasing its significant potential for application in clinical auxiliary diagnosis.

## 2 Our Proposed MFSUnet

**FIGURE 1:**
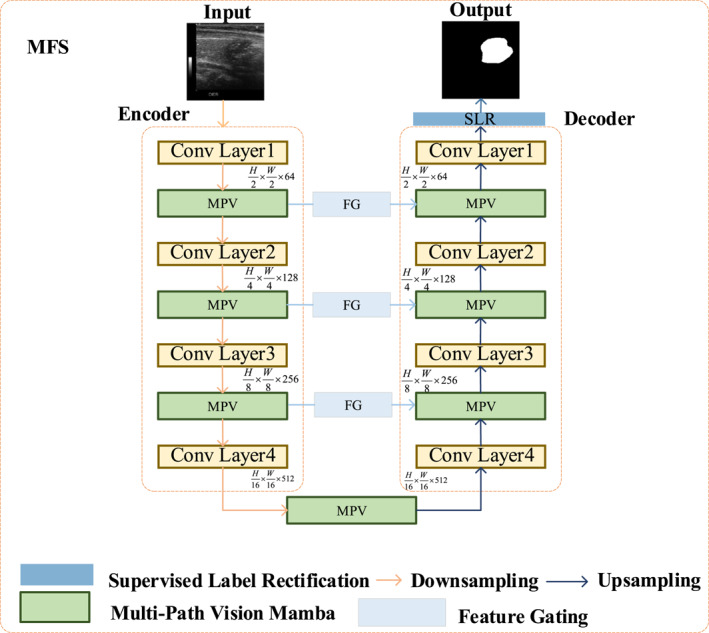
The overall architecture of our proposed MFS‐UNet. It follows an encoder‐decoder structure and incorporates our novel MPV, FG, and SLR modules to enhance segmentation performance.

To address challenges such as blurred boundaries and variable object scales in medical image segmentation, we propose a novel MFSUnet architecture, as shown in Figure 1. This network adopts a classical encoder‐decoder architecture as its backbone. The encoder consists of a series of downsampling operations (such as strided convolutions or max pooling) and feature extraction modules, responsible for mapping the input image *I* (dimensions *H* × *W* × *C*_in_) to multilevel deep semantic features {*E*_1_, *E*_2_,… *E*_*L*_}. The decoder gradually restores spatial resolution through upscaling operations (e.g., transposed convolutions) and feature fusion, ultimately generating a precise segmentation probability map *P* (dimensions *H* × *W* × *N*_class_) that matches the original image size.

Unlike traditional U‐shaped networks [18], we have embedded several innovative core modules into this framework to enhance segmentation performance. Specifically, we introduce an MPV module at each stage of the encoder and decoder to efficiently capture both local details and global dependencies of the features.

Furthermore, to optimise the information flow between the encoder and decoder, we have designed a FG module within the skip connections [19]. Adaptively it filters and enhances the features transferred from the encoder to the decoder, thereby suppressing irrelevant background noise. Additionally, we introduce a supervised label rectification (SLR) module just before the final network output. During the training process, this module uses ground‐truth label information to supervise and correct the features explicitly, guiding the model to learn a more accurate representation of the characteristics of the areas of the lesion. The following subsections will detail the design philosophy and specific implementation of these modules.

### 2.1 Multi‐Path Vision Mamba (MPV) Module

**FIGURE 2:**
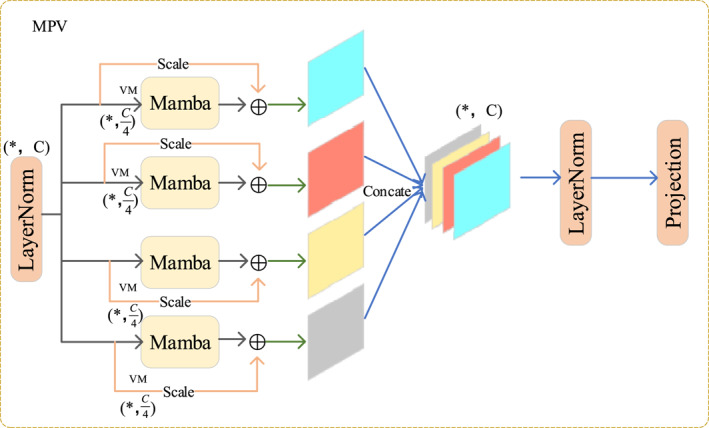
The detailed architecture of the MPV module. It processes the input feature map through four parallel Mamba blocks, each capturing dependencies in a different spatial direction, and then fuses these features to produce a comprehensive output.

To efficiently establish long‐range dependencies while preserving rich local texture information, we designed the MPV module as shown in Figure 2. It is important to clarify that this module achieves a comprehensive contextual understanding not by altering the feature map's spatial resolution, but by scanning the input from multiple directions in parallel. This multi‐perspective aggregation is key to its robust feature representation. This module is inspired by the recent success of SSMs, particularly Mamba, in the vision domain. A continuous SSM can be defined by the following linear ordinary differential equations (ODEs):

(1) $$h^{\prime }\left(t\right)=A\cdot h\left(t\right)+B\cdot x\left(t\right),$$

(2) y(t)=C·h(t),

where *h*(*t*) (dimension N) is the hidden state, *x*(*t*) (dimension D) is the input, and *y*(*t*) (dimension D) is the output. A, B, and C are transformation matrices. Mamba, through input‐dependent parameterisation and a selection mechanism, can dynamically attend to or ignore information based on the input, thereby capturing long‐range dependencies with linear complexity.

In the MPV module [20], given an input feature map X (with dimensions *H* × *W* × *C*), it first passes through a layer normalisation to stabilise the features:

(3) $$X_{\text{norm}}=\text{LayerNorm}\left(X\right).$$

Subsequently, the features are fed into four parallel branches. In each branch *i* (where *i* = 1, 2, 3, 4), we first use a VM operation to flatten the 2D feature map and scan it along different directions to capture dependencies across various spatial dimensions. The Mamba block then processes the resulting 1D sequence. To preserve the original information and facilitate gradient flow, we incorporate a residual connection and a learnable scaling factor, *β*_*i*_:

(4) $$Y_{i}=\text{Mamba}\left({V\;M}_{i}\left(X_{\text{norm}}\right)\right)+\left(\beta_{i}\cdot X_{\text{norm}}\right),$$

where *V M*_*i*_ represents the vision scanning operation of the *i*th branch, ‘+’ denotes element‐wise addition, and ‘∗’ denotes element‐wise multiplication. The outputs from the four branches, *Y*_*i*_ (with dimensions *H* × *W* × *C*), are fused via a concatenation operation to form a wider feature map:

(5) $$Y_{\text{cat}}=\text{Concat}\;\left(Y_{1},Y_{2},Y_{3},Y_{4}\right)\;\left(\text{with}\;\text{dimensions}\;H\times W\times 4C\right).$$

Finally, another layer normalisation and a linear projection layer are applied to map the channel dimension back to *C*, yielding the final output feature *Z*:

(6) $$Z=\text{Projection}\left(\text{LayerNorm}\left(Y_{\text{cat}}\right)\right)\;\left(\text{with}\;\text{dimensions}\;H\times W\times C\right).$$

Through this multi‐path design, the MPV module can comprehensively model the spatial context of the input features from multiple perspectives, thereby significantly enhancing the network's feature representation capabilities.

### 2.2 Feature Gating (FG) Module

**FIGURE 3:**
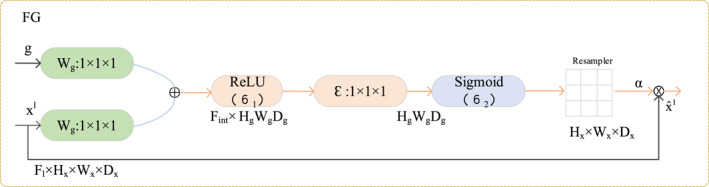
Illustration of the FG module. This module functions as an attention gate, taking a gating signal (*g*) and a feature map from the encoder's skip connection (*x* ^*l*^) as inputs. It generates an attention coefficient (*α*) to re‐weight the feature map, effectively suppressing irrelevant background information and highlighting task‐relevant features.

In U‐shaped segmentation networks, skip connections aim to combine the shallow high‐resolution features *x* ^*l*^ from the encoder with deep semantic features. To suppress potential background noise in *x* ^*l*^, we designed the FG module as shown in Figure 3, which is essentially an attention gate mechanism.

The FG module takes two inputs: the feature map *x* ^*l*^ (with dimensions *C*_*l*_ × *H* × *W*) from the *l*th layer of the encoder, and a gating signal *g* (with dimensions *C*_*g*_ × *H*′ × *W*′) from the next layer of the decoder. The gating signal *g* contains richer semantic information. The module's procedure is as follows:

First, both *x* ^*l*^ and *g* undergo separate linear transformations (implemented via 1 × 1 convolutions) and have their spatial dimensions unified. We up‐sample *g* to the same size as *x* ^*l*^, denoted as *g*_up_.

(7) $$x^{\prime }=W_{x}^{T}\cdot x^{l}+b_{x},$$

(8) $$g^{\prime }=W_{g}^{T}\cdot g_{\text{up}}+b_{g},$$

where *W*_*x*_ (with dimensions *C*_*l*_ × *C*_int_) and *W*_*g*_ (with dimensions *C*_*g*_ × *C*_int_) are the convolutional kernel weights, such that *x*′ and *g*′ both have dimensions *C*_int_ × *H* × *W*. The transformed feature maps are added element‐wise and passed through a ReLU activation function, *σ*_1_:

(9) $$F_{\text{add}}=\sigma_{1}\left(x^{\prime }+g^{\prime }\right).$$

Another 1 × 1 convolution (psi) is used to compress the channel dimension to 1, generating a compact attention activation map:

(10) $$A_{\text{act}}=\psi^{T}\;\cdot \;F_{\text{add}}+b_{\psi },$$

where *ψ* has dimensions *C*_int_ × 1.

Finally, a sigmoid activation function, *σ*_2_, maps this activation map to the range [0, 1], yielding the final attention coefficient map, *α*:

(11) $$\alpha =\sigma_{2}\left(A\;\text{act}\right)\;\left(\text{with}\;dimensions\;1\times H\times W\;\right)$$

The values in *α* reflect the importance of different spatial locations. Finally, *α* is multiplied element‐wise with the original encoder feature *x* ^*l*^ to produce the final output, ${\hat{x}}^{l}$:

(12) $${\hat{x}}^{l}=x^{l}\cdot \alpha ,$$

In this way, the FG module adaptively recalibrates the feature responses in the skip connection, enabling the model to focus more on regions relevant to the segmentation target and thereby improving segmentation accuracy.

We acknowledge the architectural similarity of our FG module to the attention gate in AttU‐Net. Its innovative value, however, lies not in the structure itself but in its synergistic role within the MFS‐Unet framework.

The core task of the FG module is to optimise the information flow in the skip connections. It provides a “purified” feature map for the subsequent MPV module, enabling it to model the global context of the target region more efficiently. Concurrently, this refined information flow improves the network's initial prediction accuracy, allowing the SLR module to focus on correcting subtle boundary issues rather than large‐scale background errors.

Therefore, the uniqueness of the FG module is its function as a critical ‘connector’ that synergistically optimises and maximises the performance of our framework's two primary innovations, the MPV and SLR modules.

### 2.3 Supervised Label Rectification (SLR) Module

**FIGURE 4:**
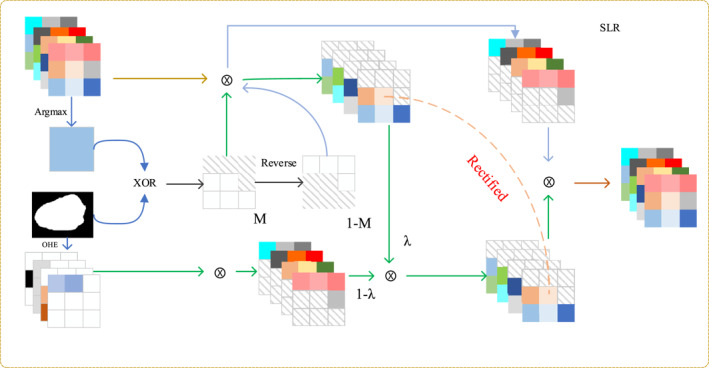
The workflow of the SLR module. This module leverages both the ground truth label and the intermediate prediction to create a rectified feature map. It identifies inconsistencies between the prediction and the label to generate a correction mask, which is then used to refine the features passed to the next decoder stage, thereby mitigating error propagation.

Conventional segmentation networks typically compute the loss function only at the final output layer, which leaves the feature learning in intermediate layers without direct and explicit supervisory signals. To introduce stronger supervision at the feature level, we innovatively propose the supervised label rectification (SLR) modules, as shown in Figure 4.

The core idea of the SLR module is to identify the regions where the model's prediction is incorrect and apply special processing to the features in these areas. Its workflow is as follows: In each training iteration, given the network's prediction probability map *P* (with dimensions *H* × *W* × *N*_class_) and the one‐hot encoded ground‐truth label *Y*_ohe_ (with dimensions *H* × *W* × *N*_class_), we first obtain the prediction mask $\hat{Y}$:

(13) $$\hat{Y}=\arg\;\;\max\;c\left(P\right)\left(\text{with}\;\text{dimensions}\;H\times W,\text{and}\;\text{values}\;\text{from}\;0\;\text{to}\;N_{\text{class}}-1\right).$$

By comparing the prediction mask $\hat{Y}$ with the ground‐truth label *Y*, we generate a boolean error region mask *M* (with dimensions *H* × *W* × 1), where a value of one indicates a pixel that was incorrectly predicted:

(14) $$M\left(i,j\right)=\left\{\begin{array}{l} 1,if\;\hat{Y}\left(i,j\right)\neq Y\;\left(i,j\right);\\ 0,otherwise \end{array}\right.$$

We partition the decoder's output feature map *F*_dec_ (with dimensions *H* × *W* × *C*_dec_) into two parts based on the error mask *M*:

(15) $$F_{\text{wrong}}=F_{\text{dec}}\cdot M,$$

(16) $$F_{\text{correct}}=F_{\text{dec}}\cdot \left(1-M\;\right).$$

To rectify the errors, we introduce a correction mechanism controlled by hyperparameters lambda and gamma. We apply a punitive transformation to the features in the erroneous regions and then recombine them with the features from the correct regions:

(17) $$F_{\text{mixed}}=\left(1-\lambda \right)\cdot F_{\text{correct}}-\lambda \cdot \gamma \cdot F_{\text{wrong}}.$$

This transformation aims to weaken the feature responses in incorrectly predicted regions while preserving the features in correct regions.

To validate the robustness of our proposed SLR module and provide experimental evidence for selecting key hyperparameters, we conducted a detailed sensitivity analysis on the two core hyperparameters in Equation (17): the weighting factor *λ* and the penalty factor *γ*. These parameters jointly control the correction intensity for features in the prediction error region, playing a crucial role in the model's final performance. Using the DDTI dataset as our experimental platform, we employed grid search to investigate how different (*λ, γ*) combinations affect model performance. Specifically, we set the search range for *λ* to {0.1, 0.3, 0.5, 0.7, 0.9} and for *γ* to {0.5, 1.0, 1.5, 2.0, 2.5}. The experimental results clearly demonstrate that the model's performance exhibits sensitivity to the selection of these two parameters. Through grid search, we found that the combination (*λ* = 0.5*, γ* = 1.0) achieved the optimal segmentation performance, with a Dice score of 77.45%.

Finally, the mixed features are integrated with the original features via a residual connection to obtain the final rectified feature, *F*_out_:

$$F_{out}=F_{dec}+F_{mixed}$$

Through the SLR module, we introduce pixel‐level supervisory information into the feature learning stage, providing the model with richer and more direct gradient signals, which leads to more robust and accurate segmentation results.

## 3 Experimental Analysis

To comprehensively validate the effectiveness and generalisation capability of our proposed framework, we conducted tests on three public thyroid ultrasound datasets, which cover thyroid nodules with different morphologies, sizes, and boundary characteristics. The specific segmentation tasks include: (1) segmentation on the DDT1 dataset, (2) segmentation on the TG3K dataset, and (3) segmentation on the TN3K dataset. We performed detailed ablation studies on these three segmentation tasks to verify the performance of our proposed framework and compared it with various SOTA (state‐of‐the‐art) segmentation networks.

### 3.1 Datasets

The data used in this study were sourced from three public thyroid ultrasound image datasets. Multiple medical centres collaboratively provided these datasets and underwent rigorous ethical review and anonymisation prior to their release. All images were screened and evaluated by experienced radiologists, ultimately forming the basis of this research.

During the data annotation phase, the boundaries of thyroid nodules in each ultrasound image were independently and manually delineated by three senior physicians to establish a precise segmentation ground truth. This meticulous process ensured the high reliability of the annotations. Considering the potential differences in imaging styles across the datasets, we uniformly resized all images to a specific resolution and applied standardised preprocessing to ensure experimental fairness. In the final consolidated datasets, DDT1 [21] contains 789 images, TG3K [22] contains 3478 images, and TN3K [22] contains 3108 images.

### 3.2 Implementation Details and Evaluation Methods

For each of the three datasets (DDTI, TG3K, and TN3K), we partitioned the data into training, validation, and testing sets. To ensure a fair evaluation and prevent data leakage, where images from the same patient might appear in multiple sets, the division was performed at the patient level. Specifically, we followed a standard 80%‐ 10%–10% split ratio. Consequently, 80% of the patients' data was allocated for model training, 10% for validation and hyperparameter tuning, and the remaining 10% was held out as a final test set for performance evaluation.

Prior to training, all images from the datasets underwent a consistent preprocessing procedure. Each image was resized to 256 × 256 pixels and normalised by scaling its pixel values to the [0, 1] range. To further enhance the diversity of the training data and improve model robustness, we employed several data augmentation strategies, including random horizontal flipping, random rotations, and random scaling. All experiments were conducted on a workstation equipped with an NVIDIA RTX 4090 GPU (24 GB VRAM), and the model was built using the PyTorch deep learning framework [23]. For model optimisation, we employed the AdamW optimiser. The entire training process ran for 300 epochs with a batch size of 8. The initial learning rate was set to 10^*−*4^, with a weight decay of 10^*−*5^. To dynamically adjust the learning rate during training, we implemented a learning rate annealing strategy, which reduces the learning rate by 50% whenever the validation loss fails to improve for 10 consecutive epochs [24].

To guide network training, we used a composite loss function combining Dice loss and binary cross‐entropy (BCE) loss. To guide network training, we used a composite loss function combining Dice loss and binary cross‐entropy (BCE) loss. We chose this combination for its complementary supervision. The BCE loss provides fine‐grained pixel‐level supervision, ensuring the model accurately learns boundary details. Meanwhile, the Dice loss offers robust region‐level supervision, which effectively handles the class imbalance common in thyroid nodule segmentation and maintains overall structural consistency. This combination allows the model to balance local details with global structure during optimization, thereby enhancing the final segmentation performance. This hybrid loss is particularly effective in addressing potential class imbalance in segmentation tasks, ensuring both region‐level and pixel‐level accuracy [25]. For quantitative assessment of segmentation accuracy, we employ evaluation metrics including Dice, IoU, precision, and SE to calculate scores between predicted segmentation maps and ground truth.

Furthermore, to directly validate our model's claimed advantage in handling blurred boundaries and achieving precise contour segmentation, we incorporated the 95% Hausdorff Distance (HD95) as an additional evaluation metric. Unlike overlap‐based metrics, HD95 specifically quantifies the maximum discrepancy between the predicted and ground‐truth contours, making it highly sensitive to boundary deviations. The calculation formulae are as follows:

(18) $$\text{Dice}=\frac{2\times \text{TP}}{2\times \text{TP}+\text{FP}+\text{FN}},$$

(19) $$\text{IoU}=\frac{\text{TP}}{\text{TP}+\text{FP}+\text{FN}},$$

(20) $$\text{Precision}=\frac{\text{TP}}{\text{TP}+\text{FP}},$$

(21) $$\text{SE}\;\left(\text{Sensitivity}\right)=\frac{\text{TP}}{\text{TP}+\text{FN}},$$

(22) $$\text{HD}95\left(A,B\right)=\max\left(h_{95}\left(A,B\right),h_{95}\left(B,A\right)\right),$$

To statistically validate the significance of our model's performance improvements, we employed a paired *t*‐test. On the test set of each dataset, we compared the sample‐wise results of our model against the second‐best performing method on key metrics. A p‐value of less than 0.05 was considered statistically significant.

In the performance evaluation of medical image segmentation, TP, FP, and FN are three core pixel‐level metrics. TP represents the target pixels correctly identified by the model; FP represents the background pixels that the model mistakenly classifies as the target, also known as a ‘false alarm’; and FN represents the actual target pixels that the model fails to identify (i.e., misclassifies as background pixels), which is referred to as a ‘miss’. Together, these three metrics quantify the accuracy and completeness of the model's prediction against the ground truth label and form the basis for calculating overlap measures such as the Dice coefficient.

### 3.3 Results and Discussion

Performance Comparison and Analysis. As shown in Table 1, our proposed network outperforms all compared existing methods on the Dice and IoU metrics across all three thyroid datasets (DDT1, TG3K, and TN3K), which powerfully demonstrates the practical effectiveness of our proposed framework. This superior performance can be primarily attributed to two key factors: first, the adoption of a stable encoder‐decoder architecture as the backbone, and second, our innovative design of the MPV, FG, and SLR modules, which significantly enhance the network's feature extraction and integration capabilities. Quantitatively, compared with the classic U‐Net baseline model, our model achieved significant Dice score improvements of 2.54%, 1.71%, and 1.97% on the DDT1, TG3K, and TN3K datasets, respectively. The success of our model highlights the superiority of its unique structural design, as even other advanced methods that also employ multi‐scale strategies fall short of our model's accuracy.

**TABLE 1:** Quantitative comparison of different models on DDT1, TG3K, and TN3K datasets, including model complexity.

Model	Params (M)↓	FLOPs (G)↓	DDT1					TG3K					TN3K				
			Dice (%)	IoU (%)	Precision (%)	SE (%)	HD95 (↓)	Dice (%)	IoU (%)	Precision (%)	SE (%)	HD95 (↓)	Dice (%)	IoU (%)	Precision (%)	SE (%)	HD95 (↓)
U‐Net [3] (2015)	7.76	15.8	74.91	62.35	81.77	96.31	8.52	95.21	96.05	96.45	97.62	4.15	77.31	67.62	80.91	81.75	7.98
U‐Net++ (2018) [26]	9.04	18.2	75.05	63.41	74.82	97.62	8.31	95.33	97.25	97.78	97.65	4.02	76.81	66.25	77.89	81.92	8.11
AttU‐Net (2021) [27]	8.95	17.5	75.68	63.49	80.99	97.65	8.15	95.91	97.08	97.61	97.08	3.88	77.63	67.19	80.55	82.76	7.53
CE‐Net (2019) [28]	29.5	48.6	72.93	60.31	75.23	97.21	8.95	95.28	96.77	98.05	98.05	4.21	78.01	67.95	81.03	82.45	7.21
DSUNet (2025) [29]	12.4	25.1	75.25	62.78	77.73	78.31	8.24	96.55	94.01	96.48	97.15	4.55	76.33	65.69	77.01	81.35	8.35
R2UNet (2018) [30]	10.8	22.4	74.18	62.45	82.01	81.23	8.66	96.61	93.42	96.19	96.75	4.41	77.12	66.98	80.34	83.41	7.82
UKAN (2024) [31]	25.6	45.2	76.82	64.45	75.68	80.01	7.14	97.91	95.55	97.43	98.35	3.15	75.03	64.32	76.38	81.88	8.54
UNext (2022) [32]	1.30	2.80	69.15	55.72	77.22	80.33	9.88	97.28	94.98	96.85	97.83	3.65	71.55	59.95	76.11	77.93	9.12
WRNet (2022) [32]	9.20	19.5	76.01	53.95	81.55	78.21	7.45	97.83	95.61	96.99	98.29	3.21	78.33	68.21	80.31	83.92	6.45
**Ours**	**5.80**	**6.50**	**77.45**	**65.11**	**83.28**	**80.92**	**6.25**	**98.02**	**96.01**	**97.59**	**98.45**	**2.89**	**79.28**	**69.34**	**82.35**	**83.01**	**5.91**

*Note:* The best results are highlighted in bold. For complexity and HD95, (↓) indicates that lower values are better.

Although a 2.54% improvement in Dice score may appear modest numerically, it holds significant clinical importance. First, higher segmentation accuracy directly impacts diagnostic precision for minute nodules. Misdiagnosis or missed detection of these small nodules remains a common clinical challenge. The improvement in Dice score indicates our model can more reliably and comprehensively capture these small targets, reducing the risk of missed diagnoses due to incomplete segmentation and providing clinicians with more dependable morphological evidence. Second, precise segmentation forms the foundation for calculating nodule volume and assessing growth changes during quantitative evaluation and follow‐up monitoring. A more accurate segmentation contour significantly reduces volume calculation errors. For instance, during 6‐ to 12‐month follow‐ups, even with minimal nodule volume changes, our model delivers more reliable tracking data. This helps physicians distinguish between genuine growth and measurement errors, enabling more precise clinical decisions—such as whether to proceed with a biopsy. Therefore, this numerical improvement is not merely a technical metric enhancement; it directly translates into higher diagnostic confidence and more precise disease management capabilities in clinical applications, bringing our research closer to real‐world clinical needs.

As shown in Table 1, our model achieved the best average performance across all key metrics. To further verify that this advantage is not due to chance, we conducted statistical significance testing.

On the DDT1 dataset, the improvement of our model's Dice score over the second‐best method was found to be statistically significant (*p <* 0.05). Similarly, on the TG3K dataset, the difference in Dice scores against the runner‐up also reached statistical significance (*p <* 0.04). For the most challenging TN3K dataset, the lead of our model in the Dice metric over the second‐best method was also statistically significant (*p <* 0.05).

These test results provide strong evidence that the architectural design of MFS‐Unet yields a substantial and statistically robust performance gain, rather than a marginal lead in mean values.

Effectiveness of the Model's Structural Design. The structural design of our model plays a crucial role in its high performance. The core MPV module, with its powerful multi‐path parallel learning capability, effectively captures the features of morphologically diverse thyroid nodules from various receptive fields and scales. This allows it to adeptly handle the challenges posed by the diversity in lesion size and shape. The FG module further enhances the model's deep semantic understanding by dynamically fusing multi‐scale features from different network levels, and it effectively alleviates the problem of imbalanced feature representation between shallow and deep layers. Furthermore, the SLR module strengthens the interaction between high‐level features and the final supervision signal during the decoding stage. It adaptively recalibrates feature weights to precisely focus the model's attention on the nodule regions while suppressing background noise interference. It is due to the synergistic effect of these three modules, each performing its specific function, that the segmentation performance for thyroid nodules, especially for challenging targets such as small or ill‐defined nodules, has been significantly improved.

**FIGURE 5:**
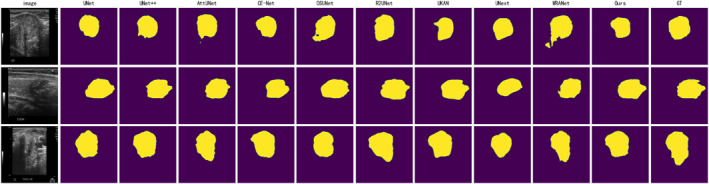
Comparison of visual segmentation results between our model (Ours) and nine other SOTA methods on the DDTI dataset. Each row represents a different test sample. From left to right: Original Image, UNet, UNet++, AttU‐Net, CE‐Net, DSUNet, R2U‐Net, UKAN, UNext, WRANet, our model, and Ground Truth (GT).

**FIGURE 6:**
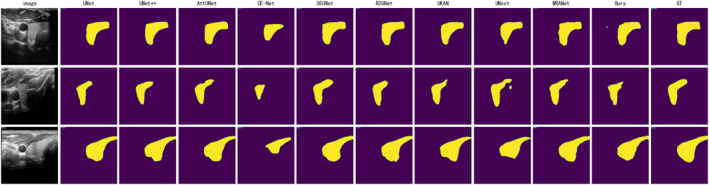
Comparison of visual segmentation results between our model (Ours) and nine other SOTA methods on the TG3K dataset. Each row represents a different test sample. From left to right: Original Image, UNet, UNet++, AttU‐Net, CE‐Net, DSUNet, R2U‐Net, UKAN, UNext, WRANet, our model, and Ground Truth (GT).

**FIGURE 7:**
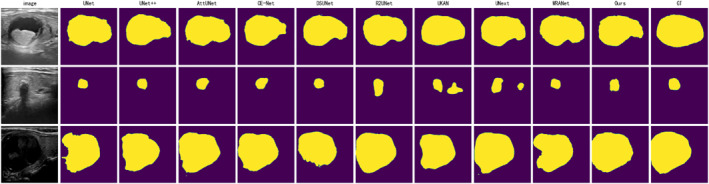
Comparison of visual segmentation results between our model (Ours) and nine other SOTA methods on the TN3K dataset. Each row represents a different test sample. From left to right: Original Image, UNet, UNet++, AttU‐Net, CE‐Net, DSUNet, R2U‐Net, UKAN, UNext, WRANet, our model, and Ground Truth (GT).

Figures 5, 6, 7 display the segmentation visualisation results of our proposed model (Ours) and nine other state‐of‐the‐art (SOTA) methods on the DDTI, TG3K, and TN3K datasets, respectively. It is visually evident from the figures that our model achieves superior segmentation performance across these three datasets, with its predicted lesion locations and contour boundaries closely matching the ground truth. In contrast, other SOTA methods frequently exhibit pixel misclassification errors, leading to degraded segmentation performance. For instance, in the first row of images from the DDTI dataset (Figure 5), the segmentation result of DSUNet [29] shows internal voids. At the same time, WRANet [33] fails to delineate the complete nodule contour accurately. Similarly, in the second row of the TG3K dataset (Figure 6), CE‐Net [28] almost completely misses the lesion area, and UNext [32] produces significant false positives (FP) above the nodule.

Beyond these examples, most other SOTA methods demonstrate similar segmentation errors. Particularly in the TN3K dataset (Figure 7), the small nodule in the second row of images poses a significant challenge for all models, with several methods, including UNet, UKAN [31], and UNext, failing to segment the lesion completely. Across all images in Figures 5, 6, 7, while most models can successfully segment the general outline of the lesion, a closer inspection reveals that other advanced methods also suffer from issues such as inaccurate segmentation regions and unclear physical boundaries. In comparison, our model can more accurately identify the lesion's structure and delineate smooth clear physical boundaries, thereby yielding more reliable visualisation results.

Although MFS‐Unet demonstrates superior overall performance, we recognise its limitations in certain extreme scenarios. For instance, when processing extremely small nodules, its low‐contrast features can easily be confused with background noise, leading to incomplete segmentation. Furthermore, when images exhibit severe acoustic shadowing artefacts, these artefacts can obscure nodule boundaries, similarly leading to undersegmentation. These cases highlight areas for future improvement, such as further enhancing the model's robustness against noise and artefacts.

### 3.4 Ablation Studies

To systematically evaluate the performance of each innovative component in our model, we conducted a detailed ablation study. The primary goal was to validate the effectiveness of our proposed MPV, FG, and SLR modules. To achieve this, we began with a baseline encoder‐decoder architecture and progressively integrated each module, observing the impact on segmentation performance across the three datasets. This step‐by‐step analysis allows us to quantify the contribution of each component to the final MFS‐Unet model. Throughout all ablation experiments, the training strategy for all models remained consistent with that of the main experiments. Below, we provide a detailed analysis of the results.

To evaluate the feature learning capability of each component, we investigated the effects of the MPV, FG, and SLR modules on the segmentation performance across our three datasets under identical training conditions and performed a comparative analysis. We sequentially added the different modules to the baseline encoder‐decoder architecture. We designate *MFS‐Unet w/o F&S* to denote the architecture without the FG and SLR modules, and *MFS‐Unet w/o S* to denote the architecture without the SLR module. The quantitative analysis results are shown in Table 2.

**TABLE 2:** Quantitative comparison of ablation studies by component on the DDTI, TG3K, and TN3K datasets.

Network	DDTI		TG3K		TN3K	
	Dice (%)	IoU (%)	Dice (%)	IoU (%)	Dice (%)	IoU (%)
Encoder‐decoder	74.91 (± 0.68)	62.35 (± 0.55)	96.31 (± 0.42)	95.21 (± 0.38)	77.31 (± 0.59)	67.62 (± 0.47)
MFS‐Unet w/o F&S (ours)	76.82 (± 0.45)	64.45 (± 0.39)	97.91 (± 0.28)	95.55 (± 0.25)	78.03 (± 0.41)	68.32 (± 0.36)
MFS‐Unet w/o S (ours)	77.15 (± 0.33)	64.88 (± 0.31)	97.98 (± 0.25)	95.89 (± 0.22)	78.89 (± 0.32)	69.05 (± 0.29)
MFS‐Unet (ours)	**77.45 (± 0.29)**	**65.11 (± 0.27)**	**98.02 (± 0.21)**	**96.01 (± 0.19)**	**79.28 (± 0.25)**	**69.34 (± 0.24)**

*Note:* The best results are highlighted in bold.

Table 2 provides a comprehensive comparative analysis of the segmentation performance of three different encoder‐decoder variants across the three datasets. Among these variants, our full MFS‐Unet achieves the most superior performance on the DDTI, TG3K, and TN3K datasets. Specifically, MFS‐Unet achieves Dice and IoU scores of 77.45% and 65.11% on the DDTI dataset, and 98.02% and 96.01% on the TG3K dataset. On all three datasets, its scores are superior to those of *MFS‐Unet w/o F&S* and *MFS‐Unet w/o S*. These results provide convincing evidence that our proposed modules effectively improve segmentation performance. However, we must also recognise that an increase in parameters and computational complexity accompanies this improvement in modelling performance.

Although our MFS‐Unet has some advantages in segmentation performance compared with its variants, it also consumes computational resources. As shown in Table 2, competitive segmentation performance was achieved across all datasets. These results highlight the robust generalisation ability of MFS‐Unet, especially in handling thyroid nodule data with different characteristics and class balances.

## 4 Conclusion

In this paper, we propose a novel medical image segmentation framework named MFS‐Unet, designed to provide an efficient solution for the precise diagnosis of thyroid nodules, thereby alleviating the workload of clinicians and enhancing diagnostic efficiency. MFS‐Unet consists of three innovative modules we have meticulously designed: the MPV module, the FG module, and the supervised label rectification (SLR) module. These components work in synergy, enabling the model to learn and understand complex features in ultrasound images from various scales and perspectives.

We conducted comprehensive quantitative and qualitative evaluations on three challenging public thyroid datasets (DDTI, TG3K, and TN3K) to validate the performance of MFS‐Unet. The experimental results demonstrate that our method outperforms current mainstream segmentation networks across all key metrics. Through detailed ablation studies, we further confirmed that each of the proposed MPV, FG, and SLR modules plays a crucial role in enhancing the final segmentation accuracy. In summary, this work reveals the excellent performance and strong generalisation ability of MFS‐Unet in the task of thyroid nodule segmentation.

Despite the significant progress achieved by our proposed model, there are still some limitations that warrant further investigation. On one hand, to enable clinical deployment on resource‐constrained portable or mobile devices, it is necessary to further reduce the model's parameter count and computational complexity in the future.

On the other hand, we believe the core modules of our model have broad potential. Specifically, the MPV module, with its ability to efficiently capture global context and long‐range dependencies at linear computational complexity, is theoretically well‐suited for tasks like large‐scale thyroid gland segmentation in CT images. The thyroid gland is often large and irregularly shaped, and MPV's global receptive field can help integrate contextual information across the entire organ, defining its borders more accurately than the limited receptive fields of traditional CNNs. For cell nuclei segmentation in pathology images, MPV could effectively model the spatial dependencies between nuclei within the large‐scale tissue microenvironment, providing contextual information for more precise diagnostic analysis.

Furthermore, the SLR module provides an effective mechanism for handling ambiguous boundaries by identifying and rectifying feature regions where model predictions and labels are inconsistent. This makes it advantageous for CT thyroid segmentation, where the boundaries between the gland and surrounding normal tissue are often indistinct. In the segmentation of densely packed nuclei in pathology images, where cells frequently overlap and have ill‐defined borders, the SLR module could guide the model to learn more robust boundary features, thus improving the accurate separation of individual nuclei.

Our future work will focus on validating and extending the potential of MFS‐Unet in these diverse clinical scenarios, aiming to support a broader range of medical image analysis tasks.

## Author Contributions

**Shaoqiang Wang:** conceptualization, methodology, software, writing – original draft. **Zhongran Liu:** data curation, validation, software. **Guiling Shi:** software, formal analysis. **Chengye Li:** data curation. **Linhao Zhang:** data curation. **Tiyao Liu:** methodology, formal analysis. **Yawu Zhao:** methodology, formal analysis. **Yuchen Wang:** validation, supervision. **Qiang Li:** supervision, project administration, writing – review editing. **Xiaochun Cheng:** supervision, project administration, writing – review editing.

## Conflicts of Interest

The authors declare no conflicts of interest.

## Data Availability

All datasets used in this study are publicly accessible.
